# Prevalence, treatment and survival of malignant peripheral nerve sheath tumor in the Danish neurofibromatosis type 1 population

**DOI:** 10.1093/oncolo/oyag255

**Published:** 2026-07-03

**Authors:** Ninna Aggerholm-Pedersen, Mette Møller Handrup, Stine Bogestofte Thomassen, Bodil Engelmann, Katja Maretty Kongstad, Maria Vad Jakobsen, Thomas Baad-Hansen, Stense Farholt, David Thomas, Cecilie Ejerskov

**Affiliations:** Department of Oncology, Sarcoma Centre of Aarhus University Hospital, Aarhus N, 8200, Denmark; Centre for Rare Diseases, Pediatrics and Adolescent Medicine, Aarhus N, 8200, Danmark; Centre for Rare Diseases, Pediatrics and Adolescent Medicine, Aarhus N, 8200, Danmark; Department of Oncology, Copenhagen University Hospital, Herlev, 2730, Denmark; Department of Pathology, Sarcoma Center of Aarhus University Hospital, Aarhus N, 8200, Denmark; Department of Pathology, Sarcoma Center of Aarhus University Hospital, Aarhus N, 8200, Denmark; Department of Orthopedic Surgery, Sarcoma Center of Aarhus University Hospital, Aarhus N, 8200, Denmark; Department of Pediatric and Adolescent Medicine, Centre for Rare Diseases, Copenhagen University Hospital—Rigshospitalet, Copenhagen, 2100, Denmark; Centre for Molecular Oncology, University of New South Wales, NSW, 2052, Australia; Department of Oncology, Sarcoma Centre of Aarhus University Hospital, Aarhus N, 8200, Denmark

**Keywords:** NF1 associated MPNST, sarcoma, prognosis

## Abstract

**Purpose:**

While neurofibromatosis type 1 (NF1)-associated malignant peripheral nerve sheath tumor (nfMPNST) is often considered to have a particularly poor prognosis compared to sporadic malignant peripheral nerve sheath tumor (sMPNST), this assumption remains insufficiently substantiated. This study aimed to compare clinical characteristics, treatment outcome, and survival between nfMPNST and sMPNST in a nationwide Danish cohort.

**Patient and method:**

We conducted a retrospective cohort study of patients with malignant peripheral nerve sheath tumor (MPNST) in Denmark from 2000 to 2020. NF1-associated cases were identified through a national NF1 registry, while sporadic cases of MPNST were extracted from the Danish Sarcoma Database. Clinical, pathological, and treatment data were supplemented by chart review and national pathology records. Survival outcomes were analyzed using Kaplan–Meier estimates and Cox proportional hazards models.

**Results:**

A total of 146 patients were included, of which 42 had nfMPNST and 104 sMPNST. Patients with nfMPNST were significantly younger at diagnosis (median 37 [min-max: 12-73] vs 58 [min-max: 5-93] years, *P* < .001) and had larger tumors (>5 cm in 88% vs 50%, *P* < .001). Wide margin resection was achieved less often in nfMPNST (35% vs 66%, *P* = .003), and adjuvant radiotherapy was more commonly administered (61% vs 36%). Five-year overall survival for localized disease was significantly lower in nfMPNST (44% vs 63%, *P *= .03), with higher recurrence rates (69% vs 49%, *P* = .049) and shorter time to recurrence (median 2.2 vs 3.4 years). Among 27 sMPNST tumors analyzed by next-generation sequencing, 5 (19%) harbored pathogenic *NF1* variants.

**Conclusion:**

nfMPNST presents at a younger age with more aggressive features and worse outcomes compared to sporadic cases. These findings support the need for tailored surveillance and treatment strategies in NF1 patients and highlight the potential role of *NF1* alterations in sMPNST pathogenesis.

Implications for Practice This nationwide, population-based study demonstrates that patients with NF1-associated malignant peripheral nerve sheath tumors (MPNST) present at a younger age and with more adverse tumor characteristics, including larger size, higher grade, and trunk location. However, multivariable analysis indicates that NF1 status itself is not an independent predictor of disease-specific mortality when established prognostic factors are considered. These findings underscore the importance of early detection and timely referral of NF1 patients with suspected malignant transformation to specialized sarcoma centers. Prognostic assessment and treatment decisions should be guided primarily by tumor-related factors rather than NF1 status alone. The identification of pathogenic NF1 alterations in a subset of sporadic MPNST further highlights the potential role of molecular profiling in selected patients and supports future evaluation of targeted therapies, including MEK inhibition, within genetically defined subgroups.

## Introduction

Malignant peripheral nerve sheath tumor (MPNST) is a rare, aggressive sarcoma type that accounts for 5%-10% of all soft tissue sarcomas.^1^^,^^2^ The prognosis for patients with MPNST is generally poor, with high mortality, and the tumors have a pronounced propensity for local recurrence and metastasis.^3^^,^^4^ MPNST can develop either as a sporadic type, after radiation therapy, or in association with neurofibromatosis type 1 (NF1), which accounts for up to 50% of the cases.^5^ The primary treatment for MPNST is radical surgery with wide margins. For high-grade and deep-seated (below the fascia) tumors, patients are treated with adjuvant radiation therapy. However, curative treatment is often not feasible due to the location or size of the tumor or the presence of distant metastases.

NF1 is a hereditary disorder resulting from a germline pathogenic variant or deletion of the *NF1* gene with a pooled estimated birth incidence fof 1 in 2.662 and a prevalence of 1 in 3.164 individuals.^6^ The life expectancy of individuals with NF1 is reduced by 8-21 years, and the most common cause of death is malignancies.^7–9^ Individuals with NF1 are at an increased risk of developing MPNST with a lifetime risk of 8-13% compared to 0.001% in the general population,^5^ and they develop MPNST at a significantly younger age.^10^ MPNST frequently develops from preexisting plexiform neurofibromas.^11^ MPNST in individuals with NF1 can be difficult to diagnose because clinical indicators of malignancy, such as pain, a growing mass, and neurologic compromise, may also be features of preexisting plexiform neurofibromas.

Reported risk factors associated with poor survival of MPNST patients include tumor size,^4^ location, tumor depth, malignancy grade, and surgical margin.^3^^,^^4^^,^^12–17^

In general, the literature on prognosis and risk factors for MPNST is sparse and conflicting, and it has yet to be resolved whether NF1-associated MPNST (nfMPNST) have a worse outcome and should be managed differently from sporadic (sMPNST).

This underscores the importance of expanding the knowledge of nfMPNST. This nationwide, longitudinal cohort study gives new knowledge on the prevalence, incidence, treatment, and survival within the nfMPNST subgroup and compares it to the sMPNST subgroup, along with an investigation of the frequency of *NF1* pathogenic gene variants in sMPNST tumor tissue, as this might offer new treatment options for patients with MPNST.

## Methods

### Patient cohorts and study design

Individuals diagnosed with sMPNST were identified via the Danish Sarcoma Database. The database contains clinical information on all sarcomas in Denmark and is a part of The Danish Clinical Quality Program (RKKP).

Individuals with NF1 and MPNST were identified through the Danish Sarcoma Database (21 individuals with NF1 and MPNST) and a nationwide project on plexiform neurofibromas (1099 individuals with NF1),^18^ resulting in a total nationwide cohort of 1120 individuals with NF1. The project was based on systematic medical record reviews of all individuals who have been or are currently monitored for NF1 at one of the two national centers of rare diseases (CRD) in Denmark. At these centers a lifelong follow-up is offered to all individuals with NF1 regardless of disease severity and socio-economic status in a free of charge healthcare system. Patients with confirmed NF1 and MPNST diagnosis were included in the nfMPNST cohort.

All patients from both cohorts were identified using the national civil registration system based on personal identification numbers. The number is unique to each person and is assigned to all permanent residents in Denmark at birth or immigration. The number is used by all public registries and links information on medical and surgical data.

Data extraction was made from the Danish Sarcoma Database, and data for both cohorts was collected from January 2000 to July 2020. Histological data extraction from the nationwide pathology-anatomical database PatoBank was used to verify and supplement clinical data on MPNST. All cases identified through the NF1 Registry were independently validated by review of histopathological reports. Incomplete reporting to the sarcoma database during the early study period explains the partial overlap between registries. Patients with neurofibromatosis type 1–associated malignant peripheral nerve sheath tumors (nfMPNST) were identified using the national NF1 Registry and cross-referenced with the institutional sarcoma database. Eleven patients were registered in both databases, while ten patients were identified exclusively through the NF1 Registry.

Due to incomplete reporting in the sarcoma database during the early study period, not all confirmed nfMPNST cases were captured. Therefore, all nfMPNST diagnoses identified through the NF1 Registry underwent independent validation through systematic review of complete medical records and histopathological reports. Only cases with confirmed pathological diagnosis of MPNST were included in the final study cohort.

NF1 status among patients with MPNST was established through a systematic retrospective review of complete medical records, including documentation from NF clinics, clinical diagnostic criteria, and genetic testing when available. Patients without evidence of NF1 were classified as sporadic MPNST.

### Variables of interest

Patient demographics: age, sex, NF1 diagnosis, cause of death, tumor size (in mm), tumor site, tumor location (superficial/below the fascia), symptoms at diagnosis (palpable mass, pain, rapid growth, neurologic deficit, or asymptomatic), malignancy grade (I/II/III), metastatic disease at the time of MPNST diagnosis, recurrence, surgical resection margin, and treatment modalities (radiation/chemotherapy).

Tumor grading in MPNST is complicated by evolving classification criteria. With the introduction of atypical neurofibromatous neoplasms of uncertain biological potential (ANNUBP), some tumors previously classified as low-grade MPNSTs would today be reclassified. All tumors in this study were diagnosed at a national sarcoma expert center, with explicit distinction between ANNUBP and MPNST, and only unequivocally malignant tumors were included.

The tumor site was categorized as head and neck, upper and lower extremities, trunk (including thorax and back), or other (abdomen, pelvis, retroperitoneal, and paravertebral). The resection margin was classified as complete resection (wide), marginal), or macroscopic residual tumor (interleasional).

### Genetic analysis of NF1 in sMPNST

A total of 65 patients were diagnosed with MPNST at Aarhus University Hospital (AUH). Formalin-fixed paraffin-embedded (FFPE) tissue blocks were available for 31 patients. Three patients were excluded since they were subsequently reclassified as probably non-MPNST. The remaining 31 cases were either not identified in the local digital pathology registry (*n* = 25) or represented revised cases in which material was retained at an external pathology department (*n* = 6). Hematoxylin and eosin (H&E) stained sections were prepared, and tumor macrodissection was performed with an estimation of tumor cell percentage. Of these, 27 patients met the eligibility criteria for next-generation sequencing (NGS) analysis.

### Sample preparation for NF1 mutation analysis

FFPE tumor tissue blocks were cut in 10 µm thick sections and collected in sterile tubes for subsequent DNA extraction. Deparaffinization was performed using xylene, and DNA was extracted using an automated QIASymphony instrument with the QIASymphony DSP DNA Mini Kit, version 1, according to the manufacturer’s protocol (Qiagen, Venlo, The Netherlands). DNA concentration was quantified using a Qubit 2.0 instrument and Qubit dsDNA HS Assay Kit according to the manufacturer’s protocol (Life Technologies Corporation).

### NGS panel sequencing and data analysis

Three microliters of DNA (concentration range 0.3-20 ng/µl) from each sample was used for NGS library preparation. Library preparation was performed using Ion AmpliSeq Library Kit 2.0 (ThermoFisher Scientific) according to the manufacturer’s protocol, although only half of the reagent volumes stated by the manufacturer for optimized reagent utilization were used. A 2-pool custom designed AmpliSeq NGS panel used for routine diagnostic procedures was applied for the NGS analysis. The custom designed AmpliSeq panel contained primers covering >95% of the coding region of NF1 (NM_001042492.2). IonXpress barcode adapters (ThermoFisher Scientific) were added for sample identification. The libraries were quantified using the Ion Library TaqMan Quantitation Kit (ThermoFisher Scientific). Following library quantification, the libraries were diluted to 40 pM and pooled before template preparation using the Ion 540 Kit-Chef (ThermoFisher Scientific) on the Ion Chef instrument (ThermoFisher Scientific). Sequencing was performed on the Ion GeneStudio S5 System (ThermoFisher Scientific), and data analysis was performed using Ion Reporter software version 5.18.4.0 (ThermoFisher Scientific), which mapped the sequencing output to the human reference genome GRCh37/hg19.

### Ethical statement

The study was conducted in accordance with the Declaration of Helsinki. The project was approved by the Central Denmark Region Committees on Health Research Ethics (approval number: 1-10-72-371-21) and registered with the institutional data protection registry (approval number: 1-16-02-233-21).

### Statistical analysis

Frequency tables are tabulated by subgroups (nfMPNST and sMPNST) for all categorical variables and compared by a chi-squared test and a Fisher exact test depending on the number of patients in each category. The median with 5 and 95 percentiles for all continuous variables are reported and compared by Wilcoxon rank-sum test. A two-sided 5% significance level is applied to all tests with 95% confidence intervals (95% CI). Overall survival and recurrence-free survival are described using Kaplan–Meier curves. Cox proportional hazard regression model was used to perform multivariate analysis. The last date of follow-up was June 01, 2025. Statistical analyses were performed using Stata 18.0 software.

## Results

Among 1120 individuals with NF1, 42 (3.8%) had MPNST. In the Danish Sarcoma Database, 104 non-NF1 individuals had MPNST; in total, data was collected on 146 patients with MPNST, including 42 nfMPNST (29%) and 104 sMPNST (71%) patients. Patients with nfMPNST were significantly younger when diagnosed with MPNST compared to the sporadic group, with a median age of 37 years and 58 years, respectively (*P < *.001). Among individuals with NF1, 3.8% developed MPNST during the 20-year observation period. The median age at the end of follow-up was 40 years for patients with NF1 and 66.5 years for patients without NF1. Consequently, this estimate does not reflect lifetime risk, as a substantial proportion of NF1 patients had not yet reached older ages at study closure, where the risk of MPNST may be higher.

No significant difference was found between the distribution of sex, although 28 patients (67%) were female in the NF1 group. Tumors were significantly larger in the NF1 group (*P *< .001), and nearly all their tumors were larger than 5 cm (88% vs 50%, *P *< .001). In both groups, tumors most commonly occurred in the lower extremities (28% vs 32%). The 'other sites’ category encompasses tumors located in the abdominal, pelvic, and retroperitoneal regions. Most patients in the NF1 group had tumor locations below the fascia (98%) compared to 74% in the sporadic group (*P *= 0.001). Patients in the NF1 group mainly presented with pain (64%), palpable mass (43%), rapid growth (26%), and neurological deficit (19%) at the time of MPNST diagnosis, whereas the sporadic group mainly presented with a palpable mass (47%) and pain (20%). Both groups presented with grade III tumors as the most frequent type but with a higher incidence in the NF1 group (78% vs 53%). Twelve NF1 patients had metastatic disease at diagnosis (29%) compared to 18 (19%) in the sporadic group (Table 1).

**Table 1:** Demographic differences of NF1 and sporadic MPNST.

	NF1-associated MPNST	Sporadic MPNST	*P*
**Number of patients (*n*)**	42	104	
**Sex (*n*, %)**			
** Female**	28 (67%)	53 (51%)	.084
** Male**	14 (33%)	51 (49%)	
**Age at MPNST diagnosis**			
** (median years, range (min-max)**	37(13-74)	58(5-93)	**<.001** ^+^
**Cause of death (*n*, %)**			
** Total**	31	66	**.001**
** MPNST**	29 (94%)	38 (58%)	
** Cancer, other**	<3	5 (8%)	
** Other causes**	<3	23 (35%)	
**Tumor size at diagnosis**			
** (mm, median [0.05; 0.95])**	100 [40; 160]	60 [20; 200]	**<.0002**
** Tumor size ≤ 5 cm (*n*, %)**	4 (10%)	40 (40 %)	**<.0001**
** Tumor size > 5 cm**	37 (88%)	52 (50%)	
** Unknown**	<3	12(12%)	
**Tumor site (*n*, %)**			
** Extremity**	17 (42%)	46 (48%)	.110
** Head/neck**	<8	11 (12%)	
** Trunk**	<8	21 (22%)	
** Other**	14(34%)	16 (17%)	
**Tumor location (*n*, %)**			
** Superficial**	<3	26 (26%)	**.001**
** Deep**	40 (98%)	73 (74%)	
**Symptoms at diagnosis^a^ (*n*, %)**			
** Palpable mass**	18 (43%)	49 (47%)	
** Pain**	27 (64%)	21 (20%)	
** Rapid growth**	11 (26%)	13 (13%)	
** Neurologic deficit**	8 (19%)	13 (13%)	
** Asymptomatic**	3 (7%)	11 (11%)	
**Malignancy grade (*n*, %)**			
** Grade I**	2 (5%)	13 (14%)	**.049**
** Grade II**	5 (12%)	22 (23%)	
** Grade III**	32 (78%)	50 (53%)	
** Unknown**	2 (5%)	10 (5%)	
**Tumor stage at diagnosis (*n*, %)**			
** Metastatic**	12 (29%)	18 (19%)	.193
** Patients treated with curative intent**	29 (71%)	76 (81%)	
**Recurrence^b^ ( *n*, %)**	20 (49%)	37 (39%)	.308

a Patients may have reported more than one symptom.

b For patients with non-metastatic disease at time of MPNST diagnosis.

+ Statistically significant values (*p* < 0.05) are marked in bold.

### Treatment

The main treatment for localized MPNST at diagnosis was surgical excision (nfMPNST 93% and sMPNST 97%), with significantly more sMPNST patients having surgical excision as the only treatment (*P = *.003). Wide margin resections were only achieved in 35% of NF1 patients compared to 66% of sporadic patients, with marginal resection as the main final surgical margin for the NF1 group (54%). Radiation was administered as adjuvant treatment to 61% in the NF1 group compared to 36% in the sporadic group. Both groups rarely received chemotherapy as treatment (21% vs 8%) (Table 2).

**Table 2:** Treatment of localized MPNST.

	NF1-associated MPNST	Sporadic	*P*
		MPNST	
**Number of patients with localized MPNST**	29 (71)	76 (81)	
**Surgery (*n*, %)**	27 (93)	74 (97)	.307
** Surgical excision only**	7 (26)	44 (59)	**.003** ^a^
**Surgical margin (*n*, %)**			
** Wide**	9 (35)	47 (66)	**.017**
** Marginal/**	14 (54)	18 (25)	
** Interlesional**	3 (12)	6 (8)	
**Radiation (*n*, %)**			
** No radiation**	11 (39)	49 (64)	**.021**
** Pre or Postoperative radiation**	17 (61)	27 (36)	
**Chemotherapy (*n*, %)**			
** No chemotherapy**	22 (79)	70 (92)	.055
** Pre or Postoperative chemotherapy**	6 (21)	6 (8)	

a Statistically significant values (*p* < 0.05) are marked in bold.

### Survival analyses and predictors for survival

**Figure 1.**
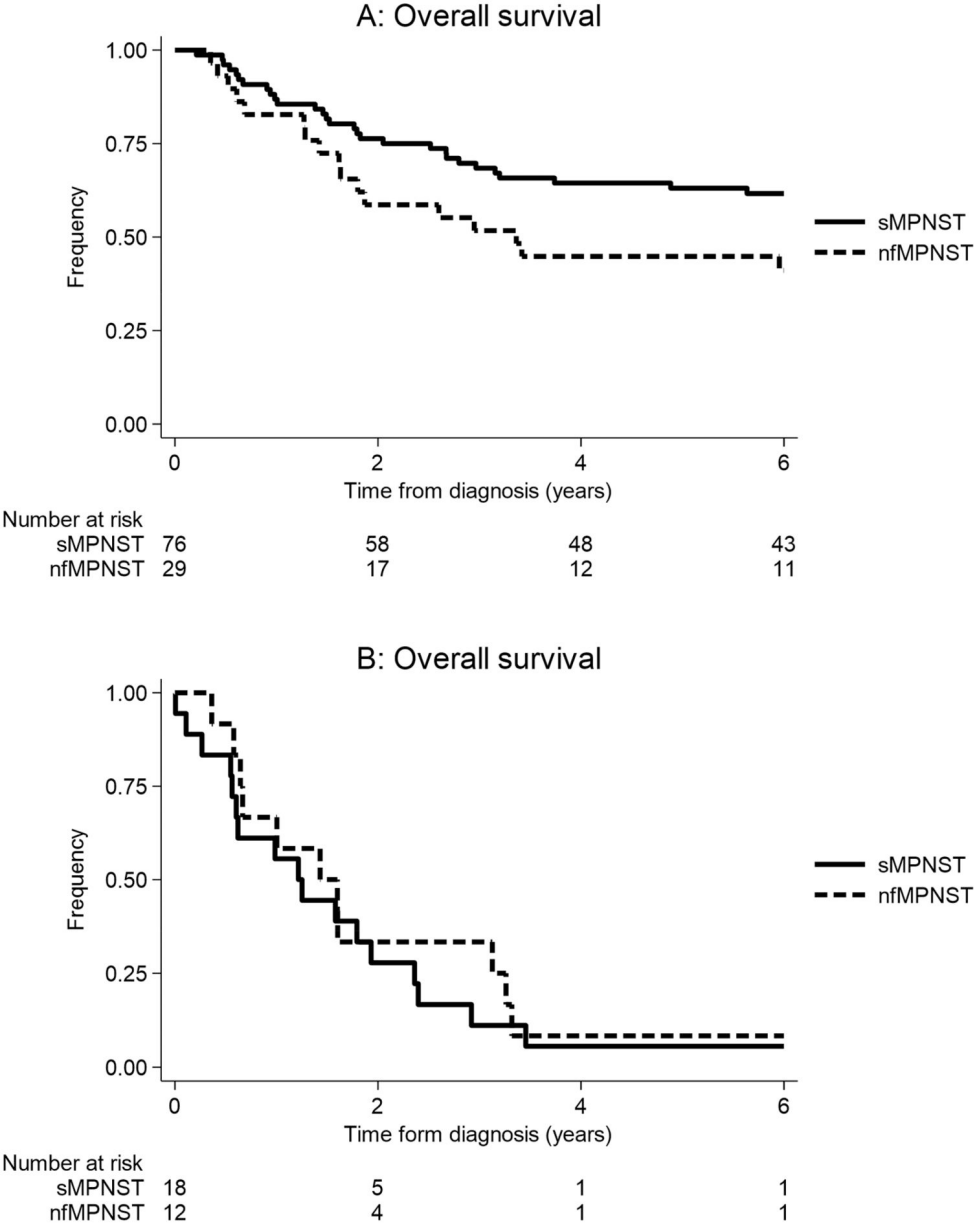
Kaplan-Meier curves for overall survival. (A) Localized disease at diagnosis; (B) Metastatic disease at diagnosis.

**Figure 2.**
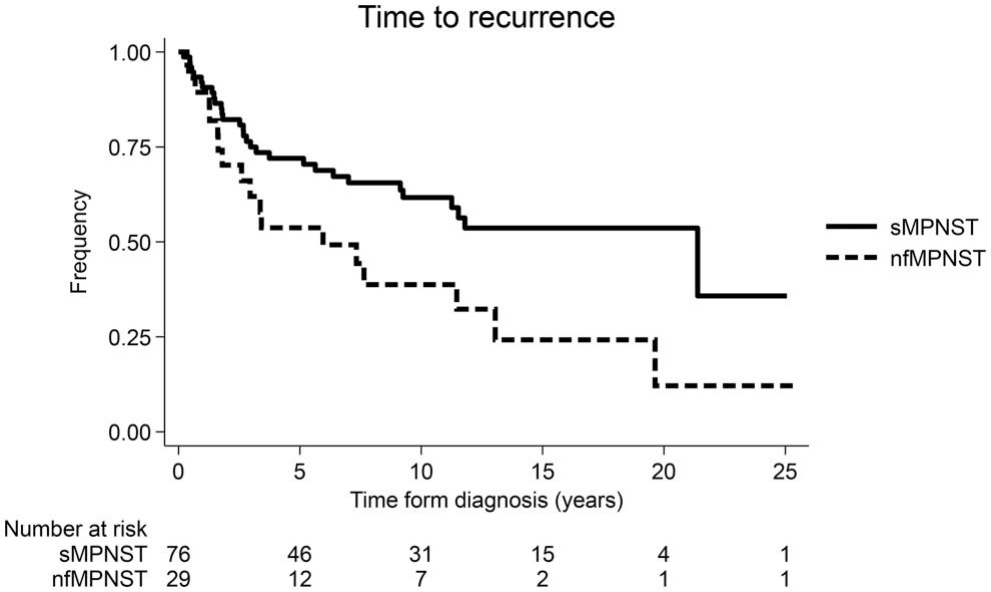
Kaplan–Meier curve for time to recurrence for localized disease at diagnosis.

**Figure 3.**
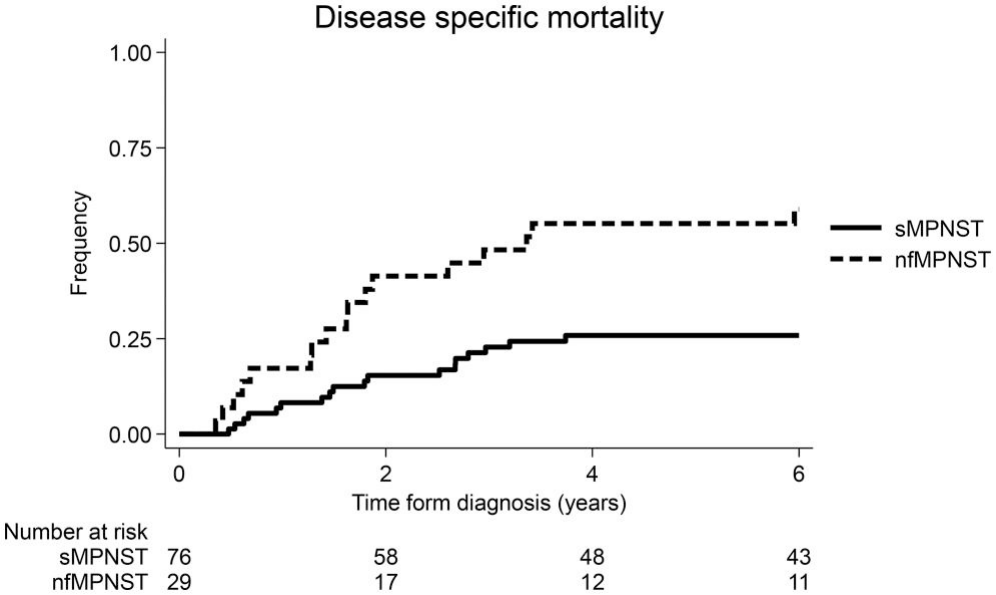
Cumulative incidence of death due to MPNST.

The median follow-up time for all patients was 3.1 years. For patients still alive at the time of inclusion, the median follow-up time was 11.1 years for the time of MPNST diagnosis. The overall survival for NF1 patients with localized disease was worse with a 2-year OS rate of 59% (95% CI, 38-74) and a 5-year OS rate of 44% (95% CI, 27-62) compared to the sporadic group with a 2-year OS rate of 76% (95% CI, 65-84) and 5-year OS rate of 63% (95% CI, 51-73) (Figure 1). In metastatic disease, the 1- and 2-year OS for nfMPNST were 67% (95% CI, 34-86) and 33 (95% CI, 10-59) with no difference to sMPNST at 1-year OS 56% (95% CI, 31-74) and 2-year OS 28% (95% CI, 10-49) (Figure 1). There was a higher frequency of recurrence in the NF1 group (69% vs 99%, *P *= 0.049) for patients with localized disease at the time of diagnosis (Table 1). The median time to recurrence for localized disease was 3.4 years for patients with sMPNST and 2.2 years for nfMPNST (Figure 2). Disease specific mortality was significantly higher for nfMPNST compared to sMPNST. By approximately 3-4 years, about 55-60% of patients in the NF1 groups have died from their disease, compared to roughly 25% in the sporatic MPNST group (Figure 3).

In the univariate analysis, patients with NF1 tumors were significantly larger at presentation and were significantly more often located in the trunk. These tumors also had a significantly higher histological grade of malignancy and marginal/interleasional margin after surgery (Table 3). In the multivariable Cox regression analysis, NF1 status was not independently associated with overall mortality (HR 1.18, 95% CI, 0.32-4.29, *P* = .81). Tumor size remained an independent prognostic factor, with tumors ≥5 cm being associated with a significantly increased risk of overall mortality (HR 3.24, 95% CI, 1.11-9.46, *P* = .032). High histological grade (grade III) was also independently associated with poorer outcome (HR 3.63, 95% CI, 1.11-11.83, *P* = .033).

**Table 3:** Univariate analysis of overall survival for localized MPNST patients.

Variable	*N*	HR	95% CI	*P*
**NF1 status**				
** No NF1**	76	1.00		
** NF1**	29	0.61	0.35-1.06	.079
**Age**				
** ≤40 years**	34	1.00		
** >40 years**	71	1.49	0.83-2.69	.191
**Sex**				
** Male**	44	1.00		
** Female**	61	0.87	0.52-1.47	.606
**Tumor size**				
** <5 cm**	40	1.00		
** >5 cm**	60	2.06	1.13-3.73	**.018** ^b^
**Tumor site**				
** Extremity**	51	1.00		
** Head/neck**	11	1.00	0.34-2.96	.986
** Trunk**	22	2.03	1.03-4.01	**.041**
**Tumor location**				
** Superficial**	23	1.00		
** Deep**	82	1.40	0.72-2.70	.318
**Malignancy grade**				
** Grade I^a^**	14	1.00		
** Grade II**	24	1.00		
** Grade III**	56	4.31	1.82-10.18	**.001**
**Surgery margin**				
** Wide**	56	1.00		
** Marginal**	32	3.01	1.65-5.50	**<.001**
** Interleasional**	9	2.27	0.85-6.08	.103

a No patient died.

b Statistically significant values (*p* < 0.05) are marked in bold.

Tumor location in the trunk was associated with an increased risk of mortality in the multivariable model (HR 3.31, 95% CI, 1.22-8.97, *P* = .018). Age showed a trend toward increased risk but did not reach statistical significance (*P* = .096). Sex and surgical margin status were not independently associated with outcome.

### Genetic analyses

A total of 65 patients with sMPNST were treated at Aarhus University Hospital. Out of those, 31 tumor samples were retrieved for genetic analysis. Four of the samples were excluded due to insufficient sample material, resulting in 27 samples eligible for NGS analysis. Table 4 shows patients with sMPNST and a pathogenic variant in the NF1 gene. A total of 5/27 sMPNST patients had a somatically acquired pathogenic variant in the NF1 gene. Four out of five patients were resented with grades II/III tumors. Patients with grade I and II tumors were treated with curative intent, whereas all patient with grade III disease were managed with palliative intent. Both patients with grade III disease and one patient with grade II disease died. All NF1 variants listed in Table 4 were reviewed for pathogenicity. The nonsense variants (p. Gln2513Ter, p. Arg461Ter, p. Arg512Ter, and p. Arg1362Ter) and the canonical splice-site variant (c.1393-1G>A) are truncating loss-of-function variants and are classified as pathogenic according to established criteria for NF1.

**Table 4:** patient with sporadic MPNST and mutation in NF1 gene of tumor tissue.

Patient ID	Dead	Malignancy grade	Treatment intention^a^	Pathogenetic NF1 variant
**1**	No	Grade II	Curative	p.Gln2513Ter (pathogenic)
**2**	Yes	Grade II	Curative	p.Arg461Ter (pathogenic)
**3**	Yes	Grade III	Palliative	p.Arg512Ter (pathogenic)
**4**	No	Grade I	Curative	c.1393-1G>A (splicesite) (pathogenic)
				p.Arg1362Ter (pathogenic)
**5**	Yes	Grade III	Palliative	p.Ser2705Asn splicesite (VUS)

Abbreviation: VUS, variant of uncertain significance.

a At diagnosis.

For tumor 4, two independent pathogenic NF1 alterations were detected in the same tumor tissue: a canonical splice-site variant (c.1393-1G>A) and a nonsense variant (p.Arg1362Ter). These represent two separate somatic hits affecting the NF1 gene.

The variant p. Ser2705Asn detected in tumor 5 is a missense variant and is currently classified as a variant of uncertain significance (VUS) based on available evidence. It is not considered pathogenic, and there is no confirmed evidence of a splice-site effect for this variant.

## Discussion

This is the first nationwide study comparing NF1-associated MPNST with sporadic MPNST. This study is populations-based including all individuals with NF1, as well as all MPNST patients registered in Denmark. A total of 3.8% in the Danish NF1 population developed nfMPNST. This study showed that nfMPNST patients had poor disease-specific survival compared to sMPNST, the patients were significantly younger and had more often larger tumors located below the fascia. Around 19% of patients with sMPNST harbored alterations in the *NF1* gene, which could potentially be targeted with Mitogen-Activated Protein Kinase (MEK) inhibitors.

The development of nfMPNST in our cohort is significantly lower than in a recent paper published by Landry et al. investigating 1607 individuals with NF1, who found that 15.1% developed MPNST.^19^ Our study is the first reporting the population-based prevalence of the development of MPNST among NF1 patients.

MPNST in general has an aggressive subset of soft tissue sarcoma, accounting for approximately 5%-10% of all soft tissue sarcoma cases. MPNST has in general a 5-year OS rate of 34%-52%, depending on factors such as tumor size, location, NF1 status, and metastatic spread at diagnosis. In contrast, other sarcoma subtypes such as liposarcoma and leiomyosarcoma often demonstrate 5-year OS rates exceeding 60%-70%.^20^

It is still debated whether NF1 serves as an independent predictor of poor prognosis in patients with MPNST. While several studies have reported no significant distinction between NF1 and sporadic patient groups,^4^^,^^21^ others indicate a poorer prognosis among NF1 patients.^12^^,^^22^^,^^23^ One meta-analysis did not find a significant difference in outcome between the two groups within the last decade due to improved survival for the NF1 patients.^24^ But a more recent meta-analysis did find NF1 to be associated with a higher risk of overall and disease-specific mortality and proposed NF1 status should be considered when staging MPNST and a closer monitoring of these patients.^25^ This study found that patients with nfMPNST who had non-metastatic disease at diagnosis had significantly worse disease-specific overall survival compared to patients with sMPNST. However, no significant difference was observed for overall survival.^25^

When grade I and II tumors were combined as low-grade MPNST, a markedly lower proportion of low-grade tumors was observed among nfMPNST compared with sMPNST (18% vs 41%). This imbalance in grade distribution likely contributes to the more favorable outcome observed in the sporadic MPNST cohort. A possible explanation is biological and clinical differences between the two entities. In NF1, malignant transformation is believed to follow a prolonged, stepwise progression from plexiform neurofibroma to ANNUBP and ultimately MPNST; consequently, surgical resection may occur relatively late in this process, potentially contributing to diagnosis at a more advanced histopathological stage. In contrast, sporadic MPNSTs may be detected earlier in their malignant course or arise through different biological mechanisms, leading to a higher proportion of lower-grade tumors.

One promising treatment is the use of MEK inhibitors, which target the RAS/MAPK signaling pathway commonly dysregulated in NF1-associated tumors. Preclinical and early clinical studies have shown encouraging results for MEK inhibitors in NF1 associated plexiform neurofibromatosis and ongoing trials are exploring their efficacy in MPNST.^26^^,^^27^ Our finding that approximately 19% of sMPNST patients also harbor pathogenic *NF1* variants suggests that MEK inhibitors may have therapeutic potential beyond the NF1 population, particularly in genetically stratified subgroups. These insights support the integration of molecular profiling into routine diagnostic workflows for MPNST, enabling the identification of patients who may benefit from targeted therapies. Furthermore, the development of biomarker-driven clinical trials is essential to evaluate the efficacy of MEK inhibitors and other targeted agents in both NF1-associated and sporadic MPNST.

A great strength of this study is that data has been collected from an unselected, nationwide patient cohort. The personal registration number system used in Denmark links all personal health information across hospitals and registries and follows a person all throughout life. It has therefore been possible to identify all individuals linked with a NF1 or MPNST diagnosis and to verify data from several sources, greatly reducing selection and referral bias. The limitations of this study should be acknowledged considering its retrospective and observational design. This includes missing data and uncertainty regarding the 21 NF1 patients identified via the Danish Sarcoma Database, who were not followed at one of the two CRD centers. While complete case ascertainment cannot be guaranteed, MPNST is typically diagnosed and treated at specialized sarcoma centers with comprehensive registry coverage. In the present study, case identification was cross-checked across multiple national registers, reducing the likelihood that NF1-associated MPNST cases were missed. We therefore consider it unlikely that any substantial under-ascertainment has occurred. Although this study is retrospective, follow-up and clinical management of patients with NF1 and MPNST in Denmark are highly centralized, as patients are typically managed either at specialized centers for rare diseases or at dedicated sarcoma centers. This centralization supports a relatively standardized follow-up and reduces variability in surveillance and treatment. Nevertheless, limitations inherent to retrospective designs remain, including potential selection bias related to patients who decline referral or follow-up, variable follow-up duration, and residual confounding from unmeasured factors. Consequently, inferences regarding natural history and prognosis should be interpreted with appropriate caution.

This nationwide, population-based study provides novel and robust evidence on the clinical characteristics and outcome of nfMPNST compared to sMPNST within the Danish population. The findings demonstrate that nfMPNST patients are diagnosed at a younger age, present with more aggressive tumor features, and experience significantly poorer disease-specific survival than their sporadic counterparts. These results support the consideration of NF1 status in prognostic assessment and clinical decision-making, and underscore the need for vigilant monitoring and tailored management strategies for NF1 patients at risk of developing MPNST. These results advocate for heightened surveillance, genetic testing, and individualized management strategies—including the potential use of MEK inhibitors—for both NF1 associated and sporadic MPNST patients. The NGS analysis was exploratory and intended to identify potential RAS/MAPK pathway alterations in sporadic MPNST that might suggest theoretical overlap with MEK inhibitor strategies used in NF1, but without implying a shared oncogenic mechanism.

## Data Availability

Due to restrictions imposed by Danish data protection legislation and the approvals obtained for this study, the data are not publicly available. The data contain sensitive personal health information and cannot be shared openly. However, de-identified data may be made available for inspection upon reasonable request to the corresponding author, subject to appropriate approvals and data protection regulations.
